# How to promote the development of a green economy: Talent or technology?—Evidence from China’s high-speed rail

**DOI:** 10.3389/fpsyg.2022.953506

**Published:** 2022-09-13

**Authors:** Dongliang Kang, Xiaoyi Zhai, Fengwen Chen, Wei Wang, Jia Lu

**Affiliations:** ^1^School of E-Commerce and Logistics Management, Henan University of Economics and Law, Zhengzhou, China; ^2^National Research Center of Cultural Industries, Central China Normal University, Wuhan, China; ^3^School of Economics and Business Administration, Chongqing University, Chongqing, China; ^4^Chongqing Jianzhu College, Chongqing, China

**Keywords:** green economy, high-speed rail, innovation, talent flow, technological update

## Abstract

The green economy is essential in supporting sustainable economic development and relies on talents and technologies. From the perspective of traditional economic theory, this study explores the impact of high-speed rail and innovation on the green economy from the perspectives of talent and technology. Using the data of 281 prefecture-level cities in China from 2008 to 2018, this study constructs empirical models to discuss the driving factors of the green economy. Empirical results show that high-speed rail and innovation can promote the development of a green economy, and the opening of high-speed rail can strengthen the positive association between innovation and a green economy. The accessibility of high-speed rail improves the flow of talent between different cities and greatly stimulates the positive impact of innovation on green economic activities. In the further test, this study explores the impact of high-speed rail and innovation on the green economy from different dimensions, including government policy, economic strength, and administrative level. During China’s 12th Five-Year Plan, high-speed rail and innovation had a positive impact on the green economy, but the impact of innovation can still be significant after this period. Moreover, the opening of high-speed rail may motivate the migration of talents from developed cities to developing ones, while developed cities can rely on technological advantages to support green economic activities. Furthermore, low-administrative level cities will rely on attracting more talents to promote a green economy due to technological disadvantages. Innovation can play a critical role in enhancing the green economy of cities with high administrative levels. Talents and technology are both important to green economic activities, and the construction of high-speed rail changes the impact of technology on the green economy through the flow of talent. Our findings can explain why the opening of high-speed rail can promote the development of a green economy and effectively help governments achieve the goal of sustainable development.

## Introduction

**FIGURE 1:**
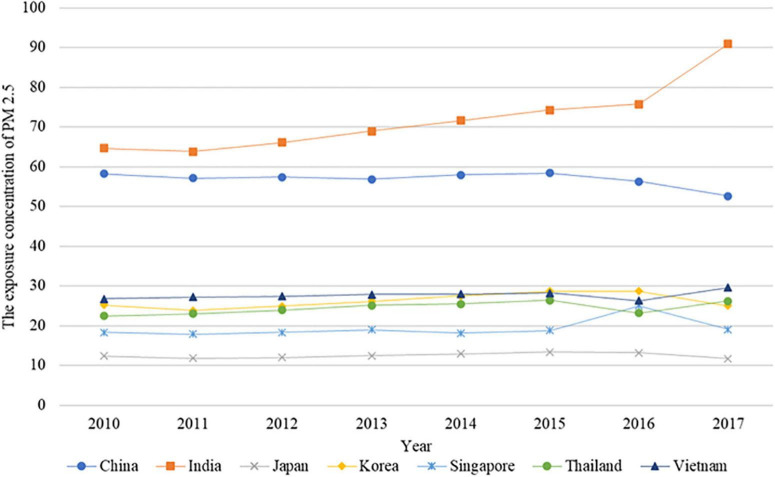
The exposure concentration of PM 2.5 in different countries.

Environmental problems caused by the emissions of pollutants have threatened the environment of human beings, and governments need to pay more attention to the environmental risks of these problems. Faced with the pressure of environmental pollution, many international organizations provide some suggestions to achieve a pathway to sustainability and set climate mitigation targets to address the climate change crisis (Allen and Craig, 2016). Although most governments recognize the harm of environmental pollution, some countries still need to rely on the output of economic activities in manufacturing industries to accelerate economies, especially in developing countries. For a long time, most developing countries preferred to accept the idea of treatment after pollution, which creates more and more environmental problems in these countries (Guo et al., 2018). As shown in Figure 1, the exposure concentration of PM 2.5 in some developing countries (India and China) is much higher than that in developed countries (Japan, Singapore, and Korea). The comparison results in Figure 1 indicate that developing countries are facing stronger pressure from environmental pollution and urgently need tools to alleviate the conflict between the environment and the economy.

To find a way out of this dilemma, the green economy, as a special concept of economic theory, was developed in response to the evaluation of environmental and social costs in regional economic development (Le Blanc, 2011). From the definition of a green economy, it is related to many dimensions of environmental protection, including low-carbon, resource-efficient, and socially inclusive (Loiseau et al., 2016). Unlike traditional economic activities, green economic activities focus on environmental and social benefits, which are the essential elements of sustainable targets. For many developing countries, a green economy can be developed by a large number of investments in key green sectors, such as the development of renewable energy and technologies in China’s 12th Five-Year Plan (2011–2015).

Promoting green industries can be seen as the industrial transformation in different regions and relies on talents and technologies. In terms of talents, productive workers can improve the quality of the labor market, which will motivate the development of skilled-intensive and clean industries (Guo et al., 2021). With the construction of high-speed rail, more and more cities can obtain some talents, especially in secondary cities. This process will improve the spatial equilibrium distribution of scientists in different regions (Dong et al., 2020). In terms of technologies, innovation outcomes can enhance the productivity of regions and play a critical role in improving resource consumption efficiency and reducing air pollutant emissions (Zhai and An, 2021). The update of technology would also determine the development of key green industries in some regions and the industrial transformation process. According to these two important factors, this study explores whether the green economy can be affected by high-speed rail and innovation.

Empirically, this study uses the panel data of 281 prefecture-level cities in China from 2008 to 2018 and examines the association between high-speed rail, innovation, and a green economy. Empirical results show that high-speed rail and innovation can promote a green economy. Moreover, the construction of high-speed rail will strengthen the impact of innovation on green economic activities. During the implementation of national policy, high-speed rail could motivate the flow of talents to be the policy-oriented migration, and its promotion to the green economy may exist in some special periods. The positive association between innovation and a green economy can be significant for a long time. Considering the economic strength of cities, developed cities can rely on technological advantages to promote the development of a green economy, while developing cities will obtain some talents to support green growth through the construction of high-speed rail. In terms of administrative level, low-administrative level cities with technological disadvantages can promote the development of a green economy by attracting more and more talents. This also supports the hypothesis that the construction of high-speed rail can shift the spatial distribution of talents.

This study provides a better understanding of the association between talent, technology, and the green economy. The main contributions are as follows: First, the flow of talents can strengthen the promotion of innovation in the green economy. The construction of high-speed rail improves the interactions between productive workers in different regions, and this will also promote knowledge creation consequences, which may have a positive impact on the green economy. Second, secondary cities connected with high-speed rail can attract more and more productive workers, and the development of green industries in these regions will be supported by the migration of talents. Reducing geographic boundaries can create a spatial equilibrium distribution of talents, especially in secondary cities with technological disadvantages. Third, innovation has played a promoting role in the development of a green economy for a long time. The outcomes of innovation can be regarded as the determining factor of green technologies, which will be the essential elements of industrial transformation.

The structure of this study is as follows: Section “Literature review and research hypotheses” provides the literature review and proposes the research hypotheses. Section “Empirical model” introduces the empirical models. Section “Empirical results” presents the empirical results. Section “Discussion” discusses the research findings based on empirical analysis. Section “Conclusion and recommendation” provides the conclusions and recommendations.

## Literature review and research hypotheses

### Green economy

A green economy can be regarded as an “umbrella” concept containing environmental advantages, which focus on the reduction of energy consumption and pollutant emissions (Loiseau et al., 2016). Unlike the traditional economic theory, the goal of a green economy is to alleviate environmental risks and ecological scarcities (Le Blanc, 2011). Based on the existing studies on the green economy, this topic includes two important dimensions: environmental benefits and social benefits (Jin et al., 2022).

In terms of the environmental dimension, green economy is a system of ideas and principles related to ecosystems and natural resources (Cato, 2012). Compared with some studies on economic development, green economy has close links with hot topics related to resource management and emission reduction (Guo et al., 2022). Guo et al. (2018) pointed out that the goal of a green economy is to resolve the conflict between economic development and environmental protection, and air pollution may be a significant factor in hindering green growth. The development of a green economy can reduce environmental pressures, and this process may be influenced by some policy-relevant factors (Lyytimäki et al., 2018). Cao et al. (2019) demonstrated that the conflict between environmental pollution and economic growth can be alleviated by innovation-driven strategies, which will also support the development of a green economy. For developing countries, governments should pay more attention to strategic sustainable development, which should be constructed by the balance between bioeconomy and the green economy the (D’Amato and Korhonen, 2021). It is worth noting that climate change may have a negative impact on the green economy in BRIC nations, and this can be explained by their economic policies and knowledge spillover (Liang and Qamruzzaman, 2022).

In terms of social dimension, the green economy the will provide socially inclusive for human beings. Loiseau et al. (2016) pointed out that industrial ecology and circular economy are the core concepts in the green economy. Liu et al. (2019) provided some interesting findings that green financial development may have a negative impact on the green economy, but these results are not significant in some developed regions. As the measurement of environmental total factor productivity is developed, a close relationship between human capital and green economic activities is proved in different fields (Zhu et al., 2020). Chen et al. (2022) also found that environmental total factor productivity can be influenced by market integration, which will be moderated by the overall development of regions. Moreover, some financial regulations—such as the rule of law, economic freedom, and inflation—could have significant causal relationship with the green economy the (Odugbesan et al., 2021). Wang et al. (2022a) demonstrated that financial inclusion would promote green economic efficiency, and this relationship should be supported by the constraints on high-polluting industries.

### High-speed rail and the green economy

The geographical mobility of talents can change the distribution of knowledge workers in different regions, and promote knowledge share in skilled individuals (Miguélez and Moreno, 2014). The construction of high-speed rail has improved the interaction between productive workers, and alleviated geographic boundaries (Dong et al., 2020). When the labor market of one area could be expended, the output of its economic activities would be promoted (Heuermann and Schmieder, 2019). In this situation, the impact of high-speed rail on economic development can be created through labor market.

For the green economy the, these special economic activities rely on productive workers, and the output of green economic activities may be determined by the number of talents (Wang et al., 2022b). Yu et al. (2022) found that high-speed rail could moderate the association between industrial transformation and green growth, and this public transportation will also improve regions’ ecology efficiency. High-speed rail will improve the reallocation of a high-skilled labor force, which can motivate the development of skilled-intensive and clean industries (Guo et al., 2021). Sun et al. (2019) found that transportation infrastructure can reduce the emissions of pollutant, and promote green growth. Yang et al. (2019) pointed out that the impact of high-speed rail on environmental pollution can be obtained through the technical effect, allocation effect and substitution effect. The construction of high-speed rail can help cities to obtain more and more talents, and also promote the development of labor market, as well as some clean industries. In this process, governments will achieve the goals of the green economy the, and obtain more and more environmental benefits. Therefore, this study proposes the following hypothesis:

**Hypothesis 1 (H1).** There is a positive association between high-speed rail and the green economy.

### Innovation and the green economy

The development of technology can improve the productivity of different regions, which will also promote the development of a regional economy (Wang et al., 2021). For the concept of the green economy the, its output will focus on environmental benefits and social benefits, while its economic benefits may be limited by the goal of sustainable development (Wang et al., 2022c). Based on the features of technological innovation, innovation outcomes could improve energy efficiency and reduce pollutant emissions, thus meeting the requirements of environmental protection (Ren and Ji, 2021). In this situation, the impact of innovation on economic development can be produced by the technological development of productivity.

Considering the definition of green economy, there is a close relationship between environmental benefits and innovation outcomes (Loiseau et al., 2016). Fernandes et al. (2021) found that sustainable technology transfer and sustainable innovations play an important role in green growth. Different outcomes of innovation may have a different impact on the green economy, and the invention patent and design patent could promote the eco-efficiency of regions, while utility patent could inhibit the green economy the (Luo et al., 2021). Ren and Ji (2021) pointed out that technological innovation can moderate the relationship between environmental regulation and the green economy. Wang et al. (2021) demonstrated that innovation can improve green total factor productivity, while this positive relationship may be limited by the development of a regional economy. From the industrial dimension, technological innovation can promote the green transformation of regions, and the spatial spillover effect of innovation will contribute to the green economy (Zhai and An, 2021). Moreover, government expenditures related to education and research and development will promote development of a green economy in developing countries, indicating that innovation has a positive impact on green economic development (Yumei et al., 2022). Therefore, this study proposes the following hypothesis:

**Hypothesis 2 (H2).** There is a positive association between innovation and the green economy.

### High-speed rail, innovation, and the green economy

The construction of high-speed rail can motivate the migration of talents, and this process will change the knowledge consequences of different regions (Dong et al., 2020). When cities connect with high-speed rail, they may obtain more and more productive workers, thus improving the outcomes of innovation (Wang and Cai, 2020). From the view of knowledge share, the flow of talents can promote the development of technology in different regions, especially in secondary cities (Sun et al., 2021).

Different from traditional economic activities, the green economy the will depend more on clean industries and innovation outcomes to reduce environmental risks (Liu and Dong, 2021). The flow of talents and the development of technology can play a critical role in improving energy efficiency and reducing pollutant emissions (Cui and Tang, 2022). Guo et al. (2021) demonstrated that the migration of talents can motivate the development of skill-intensive and clean industries, especially in cities connected with more high-speed rail. Chen (2021) found that the impact of high-speed rail on energy consumption will be realized through industrial transformation and technological update. However, there are few studies about the moderating effect of high-speed rail on the association between innovation and the green economy the, and many researchers focus on how high-speed rail can motivate the flow of talents which will support the update of technology. From this idea, the promotion of innovation in the green economy may be changed by the construction of high-speed rail through the migration of talents. Therefore, this study proposes the following hypothesis:

**Hypothesis 3.** High-speed rail can moderate the association between innovation and a green economy.

**FIGURE 2:**
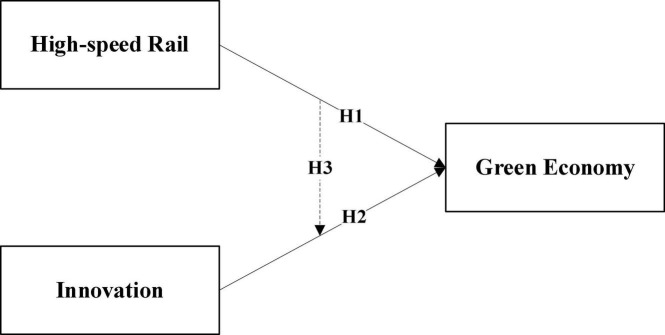
Research framework.

According to these research hypotheses, the research framework presented in this study is presented in Figure 2.

## Empirical model

### Data source

The research sample for this study comprises 281 prefecture-level cities in China from 2008 to 2018. The data of Gross Domestic Product (GDP) and innovation come from the China Urban Statistical Yearbook and Wind database. The data of air pollution, water pollution and solid pollution are obtained from the City Statistics Database of Chinese Research Data Services Platform (CNRDS). We collect the data for high-speed rail from the official railway service website of China,^1^ and obtain the opening time of high-speed rail in each city from CNRDS. The other city-level data are obtained from the China Urban Statistical Yearbook and the website of the National Bureau of Statistics of China.^2^

**FIGURE 3:**
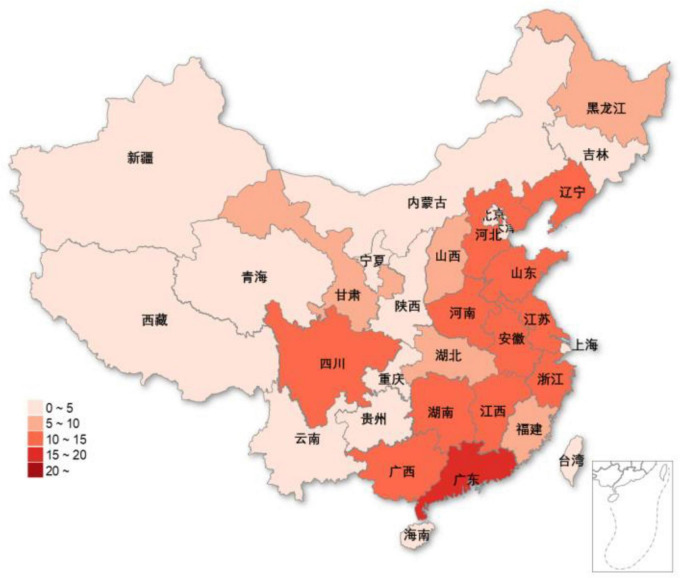
The distribution of cities connected by high-speed rails in China.

Figure 3 illustrates the distribution of cities connected by high-speed rail based on our research samples. Statistical results at the provincial level are used to show the differences between different regions, and it can be found that a large number of cities connected by high-speed rail are concentrated in the eastern region.

### Construction of variables

#### Dependent variable

Green economy represents the development of a regional economy based on environmental benefits. In terms of the definition of green economy proposed by the United Nations Environment Program, these special economic activities will focus on the importance of low-carbon, resource efficient, and socially inclusive (Loiseau et al., 2016). During China’s 12th Five Year Plan (2011–2015), local governments has paid more attention to some green sectors, such as renewable energy and environmental technology. In this situation, environmental problems may be the key factor in determining the development of a green economy, especially in developing countries. Therefore, we use the degree of environmental pollution caused by industrial manufacturing sectors to measure the green economy in regional economic activities.

To measure the degree of environmental pollution, the entropy method is used to construct the environmental pollution index of different cities. This method is a weighting method based on the dispersion degree of the evaluation index data to calculate the weights of the index (Liang et al., 2019). The specific calculation steps are as follows:

(1) standardizing the individual data in the indicator:


(1) $$x_{i,j}^{{}^{\prime }}=\frac{x_{i,j}-\text{min}\left(x_{j}\right)}{\text{max}\left(x_{j}\right)-\min\left(x_{j}\right)}$$

*x*_*i,j*_ represents the data of city i in the indicator j; max(*x*_*j*_) represents the maximum value of indicator j; min(*x*_*j*_) represents the minimum value of indicator j; $x_{i,j}^{{}^{\prime }}$ represents the standardized data of city i in the indicator j.

(2) calculating the specific gravity:


(2) $$s_{i,j}=\frac{x_{i,j}^{{}^{\prime }}}{\sum_{i=1}^{m}x_{i,j}^{{}^{\prime }}}$$

*s*_*i,j*_ represents the proportion of the indicator j’s standardized data of city i in all cities.

(3) calculating the entropy of indicator:


(3) $$e_{j}=-\frac{1}{\ln m}\sum_{i=1}^{m}s_{i,j}\ln s_{i,j}$$

*e_j_* represents the entropy value of indicator j.

(4) calculating the information utility value of *j*th indicator:


(4) $$g_{j}=1-e_{j}$$

*g_j_* represents the information utility value of indicator j.

(5) calculating the weight of *j*th indicator:


(5) $$w_{j}=\frac{g_{j}}{\sum_{j=1}^{n}g_{j}}$$

*w_j_* represents the weight of indicator j for each city.

(6) calculating the weighted value of different indicators:


(6) $$EPI_{i}=\sum_{j=1}^{n}w_{j}x_{i,j}^{{}^{\prime }}$$

*EPI*_*i*_ is the environmental pollution index of city i; *w_j_* is the weight of *j*th indicator; *m* is the number of cities; *n* is the number of indicators. The higher the value of *EPI*_*i*_, the more serious the environmental pollution of city i.

The environmental benefit of economic activities is an important part of a green economy, and how to measure the development of this special economy should incorporate the influence of environmental pollution. Referring to the method proposed by Wu and Han in 2020, the specific calculation to measure the development of a green economy at the city level is as follows (Wu and Han, 2020):


(7) $$GGDP_{i,t}=\frac{GDP_{i,t}}{EPI_{i,t}}$$

In Equation 7, *GGDP*_*i*,*t*_ is the green gross domestic product of city i in year t; *GDP*_*i*,*t*_ is the real gross domestic product of city i in year t; *EPI*_*i*,*t*_ is the environmental pollution index of city i in year t. Combined with Equations 6, 7, it can be found that *GGDP*_*i*,*t*_ will increase as the reduction of environmental pollution, and this measurement can represent the development of a green economy from the dimension of environmental protection.

#### Independent variables

High-speed rail is an important mode of public transportation and can shift the spatial equilibrium distribution of talents in different cities (Dong et al., 2020). If one city is connected with high-speed rail, its labor market will be improved by having more and more highly productive workers. Considering a link of high-speed rail between mega city and secondary city, some talents may migrate to secondary city due to the low costs of transportation and living. Based on the opening time of high-speed rail, we use *HSR*_*i*,*t*_ to represent the high-speed rail of city i in year t. When city i connects with high-speed rail in year t, *HSR*_*i*,*t*_ will be set to 1. After year t, this variable of city i will be 1 during the sample period. When city i does not connect with high-speed rail in year t, *HSR*_*i*,*t*_ will be set to 0. We treat the variable of high-speed rail as the dummy variable to explore the impact of talents on the green economy.

Innovation represents the technological development of different regions. In traditional economic theory, technology plays a critical role in regional economic activities and is an important factor in promoting the development of the economy. In terms of the outcomes of innovation, the update of technology can improve the efficiency of energy and reduce the emissions of pollutants. Based on these advantages, innovation can bring some environmental benefits to the development of a regional economy, especially in the green economy. Following Wang et al. (2021), we use the natural logarithm of patent applications to measure the innovation of different cities and explore the impact of technology on the green economy (Wang et al., 2021).

#### Control variables

Consistent with previous literature on green economy, we select some control variables to alleviate the endogenous problem caused by missing explanatory variables (Liu and Dong, 2021; Zhai and An, 2021). These control variables include industrial structure (*STRU*), governmental intervention (*GOV*), opening-up (*OPEN*), infrastructure construction (*INFRA*), population (*POP*). An industrial structure (*STRU*) represents the development of a regional industry and is calculated as the output value of the secondary industry divided by that of the tertiary industry. Governmental intervention (*GOV*) represents the government’s support for technology and is calculated as the financial expenditure of science and technology divided by public financial expenditure. Opening-up (*OPEN*) is measured by the ratio of foreign direct investment to GDP. Infrastructure construction (*INFRA*) represents the construction of a city and is measured by the natural logarithm of the road construction area. Population (POP) represents the population density of a city and is measured by the population per unit area. Table 1 provides the definitions of all variables used in the empirical analysis.

**TABLE 1:** The definitions of variables.

Variables	Type	Definition
*GGDP*	Dependent Variable	Green economy is calculated as the real GDP divided by the index of environmental pollution in Equation 7.
*HSR*	Independent Variables	High-speed rail is a dummy variable. If city i connects with high-speed rail in year t, HSR will be 1; otherwise HSR will be 0.
*LnInnovation*		Innovation is measured by the natural logarithm of patent applications.
*STRU*	Control Variables	Industrial structure is calculated as the output value of the secondary industry divided by that of the tertiary industry.
*GOV*		Governmental intervention is calculated as the financial expenditure of science and technology divided by public financial expenditure.
*OPEN*		Opening-up is measured by the ratio of foreign direct investment to GDP
*INFRA*		Infrastructure construction is measured by the natural logarithm of the road construction area.
*POP*		Population is measured by the population per unit area.

### Construction of models

To test the hypothesis proposed in Section “Literature review and research hypotheses,” we use the panel data of China’s cities to construct empirical models and explore the association between high-speed rail, innovation, and the green economy. In the basic model, the impact of *HSR* or *LnInnovation* on *GGDP* can be captured in the same year so that we set all variables in its current value, including dependent variables, independent variables, and control variables. In the further test, we will discuss the impact of *HSR* or *LnInnovation* on *GGDP* with different lags. The basic empirical model is as follows:


(8) $$\begin{array}{c} GGDP_{i,t}=\alpha_{0}+\beta_{1}HSR_{i,t}+\beta_{2}LnInnovation_{i,t}+\beta_{3}STRU_{i,t}\\ \;+\beta_{4}GOV_{i,t}+\beta_{5}OPEN_{i,t}+\beta_{6}INFRA_{i,t}\\ \;+\beta_{7}POP_{i,t}+\varepsilon \end{array}$$

In Equation 8, *GGDP*_*i*,*t*_ is the green GDP of city i in year t; *HSR*_*i*,*t*_ is whether city i connects with high-speed rail in year t; *LnInnovation*_*i*,*t*_ is the technological development of city i in year t; *STRU*_*i*,*t*_ is the industrial structure of city i in year t; *GOV*_*i*,*t*_ is the governmental intervention of city i in year t; *OPEN*_*i*,*t*_ is the opening-up of city i in year t; *INFRA*_*i*,*t*_ is the infrastructure construction of city i in year t; *POP*_*i*,*t*_ is the population density of city i in year t. ε is the error term.


(9) $$\begin{array}{c} GGDP_{i,t}=\alpha_{0}+\beta_{1}HSR_{i,t}+\beta_{2}LnInnovation_{i,t}+\beta_{3}HSR_{i,t}\\ \;\times LnInnovation_{i,t}+\beta_{4}STRU_{i,t}+\beta_{5}GOV_{i,t}\\ \;+\beta_{6}OPEN_{i,t}+\beta_{7}INFRA+\beta_{8}POP_{i,t}+\varepsilon \end{array}$$

From the viewpoint of knowledge spillover, the migration of talents can promote the technological development of different regions, indicating that high-speed rail may moderate the impact of innovation on economic activities. Therefore, we introduce the interaction term of high-speed rail and innovation (*HSR*_*i*,*t*_ = *LnInnovation*_*i*,*t*_) into Equation 8 and use Equation 9 to test the moderating role of high-speed rail.

## Empirical results

### Descriptive statistics

Table 2 reports the results of descriptive statistics for all variables. The mean of GGDP is 0.8524, and its maximum (6.4662) is great larger than its minimum (0.0867), indicating that there are great differences between China’s cities in the green economy. In terms of high-speed rail, the mean of HSR is 0.4209, which indicates that there are almost 42% cities connected with high-speed rail in the sample period. The standard deviation of innovation (1.7944) is larger than that of other variables, demonstrating that the difference in cities’ technological development is significant. It is noteworthy that the mean of innovation (6.0175) is similar to its median (5.7930), indicating that most cities can obtain some innovation outcomes in sample period. For control variables, these variables are relatively stable so that they can alleviate the endogenous problem caused by missing explanatory variables.

**TABLE 2:** The results of descriptive statistics.

Variable	Observations	Mean	Std. Dev	Min.	Median	Max.
*GGDP*	2,554	0.8524	0.9639	0.0867	0.5708	6.4662
*HSR*	2,554	0.4209	0.4938	0.0000	0.0000	1.0000
*LnInnovation*	2,554	6.0175	1.7944	2.4849	5.7930	10.4554
*STRU*	2,554	1.3809	0.5614	0.3272	1.2895	3.4381
*GOV*	2,554	0.0164	0.0188	0.0000	0.0113	0.1296
*OPEN*	2,554	0.0230	0.0222	0.0001	0.0162	0.1091
*INFRA*	2,554	6.9627	1.2256	0.0000	6.9368	9.3801
*POP*	2,554	0.9825	0.7906	0.0787	0.7677	4.2072

Table 3 reports the results of Pearson correlation matrix for all variables. The correlation coefficient between *HSR* and *GGDP* is 0.265, significant at the 1% level, indicating that there may be a positive association between high-speed rail and the green economy. The correlation coefficient between *LnInnovation* and *GGDP* is 0.390, significant at the 1% level, which indicates that there may be a positive association between innovation and the green economy. The results of *HSR* and *LnInnovation* can provide some evidence that high-speed rail or innovation may promote the development of a green economy. Moreover, the absolute correlation coefficients between control variables and *GGDP* are less than 0.6, demonstrating that these variables can effectively explain the green economy. Considering the problem of multi-collinearity, we use the VIF method to explore the relationship between independent variables or control variables and dependent variables. Based on the VIF values of all variables, it can be found that the variables selected in empirical models are not influenced by the problem of multi-collinearity.

**TABLE 3:** The results of the correlation matrix.

	*GGDP*	*HSR*	*LnInnovation*	*STRU*	*GOV*	*OPEN*	*INFRA*	*POP*	VIF
*GGDP*	1								
*HSR*	0.265***	1							1.41
*LnInnovation*	0.390***	0.527***	1						2.26
*STRU*	–0.259***	–0.261***	–0.328***	1					1.17
*GOV*	0.263***	0.223***	0.420***	–0.115***	1				1.23
*OPEN*	0.091***	0.155***	0.249***	–0.0280	0.186***	1			1.13
*INFRA*	0.246***	0.265***	0.563***	–0.186***	0.226***	0.208***	1		1.49
*POP*	0.062***	0.160***	0.247***	0.074***	0.071***	0.255***	0.213***	1	1.16

*** represent the significance at the level of 1%. Pearson correlations among variables are below the diagonal.

### Baseline test

Based on the results of correlation and VIF, *HSR* or *LnInnovation*, and control variables can explain *GGDP*, and our empirical models proposed in Equations 8, 9 will explore the association among high-speed rail, innovation, and the green economy. To obtain appropriate empirical results, the first step of baseline test will discuss the relationship between *HSR* or *LnInnovation* and *GGDP* without introducing control variables. Second, we will introduce control variables to test the impact of *HSR* or *LnInnovation* on *GGDP*. Third, the impact of *HSR* and *LnInnovation* will be considered in baseline test at the same time. Finally, the interaction term of *HSR* and *LnInnovation* will be introduced into the empirical model, and the moderating role of HSR will be tested in the baseline test. The regression results of baseline test are reported in Table 4.

**TABLE 4:** The regression results of the baseline test.

Variables	*GGDP*					
	(1)	(2)	(3)	(4)	(5)	(6)
*HSR*	0.3353***		0.1673***		0.1083**	0.0833*
	(7.67)		(4.33)		(2.53)	(1.80)
*LnInnovation*		0.1787***		0.1096***	0.1011***	0.0911***
		(9.44)		(4.67)	(4.08)	(3.93)
HSR*LnInnovation						0.1039***
						(3.11)
*STRU*			–0.3351***	–0.3112***	–0.3058***	–0.2902***
			(–7.93)	(–6.78)	(–6.85)	(–6.32)
*GOV*			9.1325***	7.1314***	7.0701***	6.9996***
			(5.10)	(3.63)	(3.61)	(3.58)
*OPEN*			1.2956	0.5198	0.3358	0.4233
			(1.52)	(0.56)	(0.37)	(0.47)
*INFRA*			0.0880***	0.0373**	0.0371**	0.0337**
			(4.66)	(2.50)	(2.48)	(2.35)
*POP*			0.0393*	0.0138	0.0086	0.0113
			(1.92)	(0.73)	(0.45)	(0.60)
*Constant*	0.5363***	–0.3818***	0.1406	–0.0122	0.0231	0.0205
	(13.30)	(–3.43)	(0.91)	(–0.08)	(0.14)	(0.12)
Year	Yes	Yes	Yes	Yes	Yes	Yes
Region	Yes	Yes	Yes	Yes	Yes	Yes
Observations	2554	2554	2554	2554	2554	2554
R^2^	0.1433	0.1964	0.2266	0.2372	0.2391	0.2455

T statistics are in parentheses; ***, **, * represent the significance at the level of 1, 5, and 10% respectively.

In Table 4, Columns (1) and (3), respectively, test the impact of high-speed rail on the green economy. Based on the results of Columns (1) and (3), the coefficient of *HSR* is 0.3353 (0.1673), significant at the 1% level, indicating a positive association between high-speed rail and the green economy the, which supports H1. Columns (2) and (4), respectively, test the impact of innovation on the green economy. Based on the results of Columns (2) and (4), the coefficient of *LnInnovation* is 0.1787 (0.1096), significant at the 1% level, indicating a positive association between innovation and the green economy the, which supports H2. From the results of Columns (1–4), the impact of high-speed rail or innovation on the green economy is still significant after introducing control variables. Column (5) tests whether both high-speed rail and innovation will influence the development of a green economy. Based on the results of Column (5), the coefficients of *HSR* and *LnInnovation* are 0.1083 and 0.1011, significant at the 5 and 1% level, indicating that high-speed rail and innovation can both promote the development of a green economy. Column (6) tests the moderating role of high-speed rail in the association between innovation and the green economy. In Column (6), the coefficient of the interaction term (HSR*LnInnovation) is 0.1039, significant at the 1%, and the coefficients of *HSR* and *LnInnovation* are still significant. The result of Column (6) can demonstrate that high-speed rail can strengthen the impact of innovation on the green economy, which supports H3.

### Heterogeneity test

According to the regression results of the baseline test, it can verify that there is a significant association among high-speed rail, innovation, and the green economy. Considering the characteristics of China’s economic development, the economic activities of different cities can be determined by some national policies, and the economic strength and administrative level of cities may also have an impact on the route of regional economic development (Cao et al., 2019; Liu and Dong, 2021; Chen et al., 2022). In this situation, we carry out an analysis of heterogeneity from three dimensions, including government policy, economic strength, and the administrative level.

#### The test of government policy

In China’s 12th Five-Year Plan (2011–2015), the government has devoted large number of resources to some green sectors. During this period, the output of green economic activities attracted the attention of local governments, and it also became the important criteria for political competition. Because this special government policy can determine the development planning of different cities, we use the time of China’s 12th Five-Year Plan to test the impact of government policy on the green economy. Specifically, the year of 2015 are selected to divide the research sample: the subsample of before 2015 and the subsample of after 2015. The empirical models constructed by Equations 8, 9 are still used in this heterogeneity test. The regression results of government policy are reported in Table 5.

**TABLE 5:** The regression results of government policy.

	*GGDP*			
	Before 2015		After 2015	
	(1)	(2)	(3)	(4)
*HSR*	0.1811***	0.1625***	–0.0599	–0.0691
	(3.795)	(3.174)	(–0.665)	(–0.652)
*LnInnovation*	0.0573*	0.0616*	0.1481***	0.1409***
	(1.675)	(1.729)	(3.311)	(3.977)
HSR*LnInnovation		0.1145***		0.0206
		(2.669)		(0.303)
*STRU*	–0.2691***	–0.2492***	–0.5192***	–0.5136***
	(–5.671)	(–4.980)	(–3.539)	(–3.388)
*GOV*	13.1397***	12.0777***	5.9767**	5.9694**
	(3.558)	(3.243)	(2.540)	(2.529)
*OPEN*	–0.5005	–0.3377	0.3494	0.2918
	(–0.505)	(–0.351)	(0.162)	(0.134)
*INFRA*	0.0336*	0.0283	0.0493**	0.0487**
	(1.666)	(1.454)	(2.358)	(2.348)
*POP*	0.0049	0.0044	0.0618	0.0616
	(0.245)	(0.224)	(1.123)	(1.120)
*Constant*	0.1607	0.1072	–0.0573	–0.0105
	(0.866)	(0.540)	(–0.145)	(–0.032)
Year	Yes	Yes	Yes	Yes
Region	Yes	Yes	Yes	Yes
Observations	1962	1962	592	592
R^2^	0.2800	0.2882	0.1525	0.1526

T statistics are in parentheses; ***, **, * represent the significance at the level of 1, 5, and 10% respectively.

In Table 5, Columns (1) and (2) test the impact of high-speed rail and innovation on the green economy before 2015. In Column (1), the coefficients of *HSR* and *LnInnovation* are 0.1811 and 0.0573, significant at the 1 and 10% level, indicating that high-speed rail and innovation can promote the development of a green economy during China’s 12th Five-Year Plan. In Column (2), the coefficient of the interaction term (HSR*LnInnovation) is 0.1145, significant at the 1% level, and the coefficients of *HSR* and *LnInnovation* are also significant. The result of Column (2) can demonstrate that high-speed rail can strengthen the impact of innovation on the green economy so that the flow of talents could moderate the promotion of technological development to green economic activities during the implementation of government policy. Columns (3) and (4) test the impact of high-speed rail and innovation on the green economy after 2015. In Column (3), the coefficient of *LnInnovation* is 0.1481, significant at the 1% level, while the coefficient of *HSR* is not significant. In Column (4), the coefficient of *LnInnovation* is 0.1409, significant at the 1% level, while the coefficients of *HSR* and interaction term are not significant. Combined with the results of Columns (3) and (4), it can be found that the development of technology will promote the green economy the after China’s 12th Five-Year Plan, but the flow of talents may not have a significant impact on green economic activities, as well as the moderating role of high-speed rail.

#### The test of economic strength

The economic strength of cities can represent the outputs of economic activities in different regions. Some China’s developed cities, such as Shanghai and Shenzhen, own obvious advantages in talent and technology, and these cities can easily achieve the goals of the green economy the, thus obtain environmental benefits and social benefits. With the continuous construction of high-speed rail, more and more talents may migrate to some developing cities due to the low costs of transportation and living (Dong et al., 2020). To explore the impact of cities’ economic strength on the green economy the, we use the per capita GDP of cities to divide the research sample, namely the subsample of developed city and the subsample of developing city. The empirical models constructed by Equations 8, 9 are still used in this heterogeneity test. The regression results of economic strength are reported in Table 6.

**TABLE 6:** The regression results of economic strength.

	*GGDP*			
	Developed city		Developing city	
	(1)	(2)	(3)	(4)
*HSR*	–0.0964	–0.2828***	0.1857***	0.1831***
	(–1.58)	(–3.98)	(3.07)	(3.09)
*LnInnovation*	0.3024***	0.2566***	–0.0447	–0.0457
	(10.72)	(9.90)	(–1.56)	(–1.58)
HSR*LnInnovation		0.2666***		–0.0456
		(5.56)		(–1.02)
*STRU*	–0.2739***	–0.2588***	–0.2694***	–0.2746***
	(–4.24)	(–4.10)	(–5.01)	(–4.99)
*GOV*	2.3509	1.9261	7.5908***	7.4445***
	(0.87)	(0.73)	(2.86)	(2.81)
*OPEN*	–4.3806***	–4.6481***	3.9836***	3.9285***
	(–3.41)	(–3.82)	(3.06)	(3.05)
*INFRA*	0.0352*	0.0276	0.0577***	0.0587***
	(1.75)	(1.54)	(2.81)	(2.84)
*POP*	–0.0079	0.0251	0.0381*	0.0400*
	(–0.19)	(0.61)	(1.80)	(1.89)
*Constant*	–0.9786***	–0.7251***	0.4594**	0.4911**
	(–4.41)	(–3.58)	(2.53)	(2.49)
Year	Yes	Yes	Yes	Yes
Region	Yes	Yes	Yes	Yes
Observations	929	929	1611	1611
R^2^	0.3647	0.3952	0.1842	0.1852

T statistics are in parentheses; ***, **, * represent the significance at the level of 1, 5, and 10% respectively.

In Table 6, Columns (1) and (2) test the impact of high-speed rail and innovation on the green economy in developed cities. In Column (1), the coefficient of *LnInnovation* is 0.3024, significant at the 1% level, while the coefficient of HSR is not significant. In Column (2), the coefficient of the interaction term (HSR*LnInnovation) is 0.2666, significant at the 1% level, and the coefficients of *HSR* and *LnInnovation* are -0.2828 and 0.2566 respectively, significant at the 1% level. The results of Column (1) and Column (2) can support that the construction of high-speed rail may motivate the migration of talents from mega cities to secondary cities, and this will limit the development of a green economy in developed cities. Furthermore, the flow of talents could moderate the promotion of technological development to green economic activities in these cities. Columns (3) and (4) test the impact of high-speed rail and innovation on the green economy in developing cities. In Column (3), the coefficient of HSR is 0.1857, significant at the 1% level, while the coefficient of *LnInnovation* is not significant. In Column (4), the coefficient of *HSR* is 0.1831, significant at the 1% level, while the coefficients of *LnInnovation* and the interaction term are not significant. Combined with the results of Columns (3) and (4), it can be found that the flow of talents will promote the green economy the in developing cities, but the development of technology may not have a significant impact on green economic activities.

#### The test of administrative level

The administrative level of cities can represent their importance in the political dimension. China’s cities can be classified into three categories, namely centrally-administered municipality, sub-provincial city and prefecture-level city. For centrally-administered municipalities and sub-provincial cities, these cities have obvious political advantages, and can obtain the strong support of the central government. However, prefecture-level cities can only get some support from provincial governments, and they may be limited by the development of a regional economy. To explore the impact of cities’ administrative level on the green economy the, we divide the research sample into two subsamples: prefecture-level cities and non-prefecture-level cities. The empirical models constructed by Equations 8, 9 are still used in this heterogeneity test. The regression results of administrative level are reported in Table 7.

**TABLE 7:** The regression results of administrative level.

	*GGDP*			
	Prefecture-level city		Non-prefecture-level city	
	(1)	(2)	(3)	(4)
*HSR*	0.0943**	0.0984**	0.0417	–1.1567*
	(2.15)	(2.17)	(0.17)	(–1.79)
*LnInnovation*	0.0335	0.0353	0.6765***	0.5104***
	(1.36)	(1.49)	(3.89)	(2.77)
HSR*LnInnovation		–0.0346		0.5293*
		(–1.09)		(1.74)
*STRU*	–0.1915***	–0.1942***	–1.7625***	–1.5679***
	(–4.48)	(–4.48)	(–3.67)	(–3.19)
*GOV*	4.7356**	4.7523**	18.0195**	18.2341**
	(2.53)	(2.54)	(2.11)	(2.21)
*OPEN*	1.0037	0.9619	–1.0105	–1.9739
	(1.15)	(1.12)	(–0.26)	(–0.50)
*INFRA*	0.0174	0.0175	0.0312	0.0238
	(1.22)	(1.20)	(0.69)	(0.55)
*POP*	–0.0044	–0.0050	0.0402	0.0701
	(–0.27)	(–0.30)	(0.22)	(0.39)
*Constant*	0.3334**	0.3478**	–3.5137**	–1.8564
	(2.09)	(2.09)	(–2.37)	(–1.18)
Year	Yes	Yes	Yes	Yes
Region	Yes	Yes	Yes	Yes
Observations	2363	2363	191	191
R^2^	0.1587	0.1595	0.5034	0.5123

T statistics are in parentheses; ***, **, * represent the significance at the level of 1, 5, and 10% respectively.

In Table 7, Columns (1) and (2) test the impact of high-speed rail and innovation on the green economy in prefecture-level cities. In Column (1), the coefficient of *HSR* is 0.0943, significant at the 5% level, while the coefficient of *LnInnovation* is not significant. In Column (2), the coefficient of *HSR* is 0.0984, significant at the 5% level, and the coefficients of *LnInnovation* and the interaction term (HSR*LnInnovation) are not significant. The results of Column (1) and Column (2) can support that the construction of high-speed rail can help prefecture-level cities to obtain some talents from mega cities, and this will promote their green economic development. Columns (3) and (4) test the impact of high-speed rail and innovation on the green economy in non-prefecture-level cities. In Column (3), the coefficient of *LnInnovation* is 0.6765, significant at the 1% level, while the coefficient of *HSR* is not significant. In Column (4), the coefficient of *LnInnovation* is 0.5104, significant at the 1% level, and the coefficients of *HSR* and interaction term are -1.1567 and 0.5293, respectively, significant at the 10% level. Combined with the results of Columns (3) and (4), the construction of high-speed rail may promote the flow of talents from mega cities to secondary cities, and this process can also strengthen the impact of innovation on the green economic activities.

### Further test

According to the construction of high-speed rail, the cities connected with this special public transportation can obtain long-term benefits from the spillover of knowledge. Moreover, the outcomes of innovation will promote the development of a regional economy in a long time, and motivate the growth of a green economy (Khan et al., 2022). In this situation, we further explore the association among high-speed rail, innovation and the green economy with considering different lag years (Yumei et al., 2022). The regression results of different lag years are reported in Table 8.

**TABLE 8:** The regression results of different lag years.

	*GGDP*					
	t–1		t–2		t–3	
	(1)	(2)	(3)	(4)	(5)	(6)
*HSR*_*t*–1_	0.1254**	0.0965*				
	(2.55)	(1.84)				
*LnInnovation*_*t*–1_	0.1168***	0.1073***				
	(4.13)	(3.94)				
HSR*LnInnovation_*t*–1_		0.1165***				
		(3.03)				
*HSR*_*t*–2_			0.1613***	0.1272**		
			(2.95)	(2.22)		
*LnInnovation*_*t*–2_			0.1282***	0.1218***		
			(4.15)	(4.00)		
HSR*LnInnovation_*t*–2_				0.1258***		
				(2.89)		
*HSR*_*t*–3_					0.1772***	0.1396**
					(2.79)	(2.11)
*LnInnovation*_*t*–3_					0.1528***	0.1508***
					(4.53)	(4.47)
HSR*LnInnovation_*t*–3_						0.1332**
						(2.54)
*STRU*	–0.3411***	–0.3288***	–0.3591***	–0.3508***	–0.3582***	–0.3534***
	(–6.44)	(–6.13)	(–5.96)	(–5.79)	(–5.11)	(–5.03)
*GOV*	7.0360***	6.9795***	7.4470***	7.4649***	6.8709***	7.0925***
	(3.11)	(3.09)	(3.18)	(3.20)	(3.18)	(3.30)
*OPEN*	0.2213	0.3491	0.1385	0.2936	–0.0020	0.1778
	(0.22)	(0.34)	(0.12)	(0.26)	(–0.00)	(0.15)
*INFRA*	0.0321**	0.0294*	0.0268*	0.0240	0.0127	0.0085
	(1.99)	(1.94)	(1.65)	(1.57)	(0.82)	(0.58)
*POP*	0.0097	0.0134	0.0116	0.0163	0.0089	0.0147
	(0.45)	(0.62)	(0.47)	(0.67)	(0.32)	(0.54)
*Constant*	0.0144	0.0034	0.1335	0.1067	0.4190*	0.3752
	(0.08)	(0.02)	(0.63)	(0.50)	(1.73)	(1.52)
Year	Yes	Yes	Yes	Yes	Yes	Yes
Region	Yes	Yes	Yes	Yes	Yes	Yes
Observations	2124	2124	1885	1885	1640	1640
R^2^	0.2304	0.2380	0.2240	0.2324	0.1971	0.2056

T statistics are in parentheses; ***, **, * represent the significance at the level of 1, 5, and 10% respectively.

In Table 8, Columns (1) and (2) test the impact of high-speed rail and innovation on the green economy with a one-year lag. Based on the results of Column (1) and Column (2), the coefficients of *HSR* and *LnInnovation* are significant, and the coefficient of the interaction term (HSR*LnInnovation) is also significant. Columns (3) and (4) test the impact of high-speed rail and innovation on the green economy with a two-year lag, and the coefficients of *HSR*, *LnInnovation* and the interaction term (HSR*LnInnovation) are still significant. Columns (5) and (6) test the impact of high-speed rail and innovation on the green economy, with a three-year lag, the results of independent variables are consistent with those of other columns. Combined with the results of Columns (1)-(6), it can be found that the impact of high-speed rail and innovation on the green economy can exist for many years, and the flow of talents and the development of technology can be regarded as the critical factors in promoting green economic growth.

## Discussion

Green economy is an important concept in traditional economic theory and has always been related to some environmental and social topics. Compared with the output of economic activities, the green economy the focuses on environmental benefits, such as improving the efficient of resource and reducing the emission of pollutant. For many developing countries, there is a heated argument about whether they should protect the environment by limiting the development of a regional economy, or ignore the environment by promoting the output of economic activities (Wang et al., 2022c). Faced with serious environmental problems, governments need to design new development strategies to achieve the goal of sustainability and resolve the conflict between environmental protection and economic development. In this situation, exploring the driving factors of a green economy has become an important step in the process of sustainable economic development.

Based on the empirical results reported in Section “Empirical results,” high-speed rail and innovation have a positive impact on the green economy, demonstrating that the flow of talents and the development of technology can both promote the output of green economic activities. Considering the influence of national development strategy, the flow of talents can become the policy-oriented migration, and the promotion of high-speed rail to the green economy the may exist in some special periods, which supports the findings of Yang et al. (2019). Similar to Dong et al. (2020), the construction of high-speed rail will motivate some talents to leave mega cities, namely developed or high administrative-level cities (Dong et al., 2020). As with knowledge creation consequences, the outcomes of technological innovation can be improved by the construction of high-speed rail, and this is consistent with Cui and Tang (2022). It is noteworthy that the moderating role of high-speed rail only exists in China’s high-level cities, which may be due to the technological and talent advantages of such cities.

## Conclusion and recommendation

### Conclusion

The conflict between environmental protection and economic development has restricted the output of regional economic activities, especially in developing countries. Green economy, as a special kind of economic concept, can provide some sustainable development strategies for governments, and its output focuses on environmental and social benefits. Consistent with traditional economic theory, the factors of labor and technology play a critical role in the development of a regional green economy. In terms of labor factor, the construction of high-speed rail can motivate the flow of talents, and help secondary cities to obtain some highly productive workers. In terms of technology factors, the outcomes of technological development will have an impact on energy consumption and pollutant emissions, which could help local governments achieve green goals. In this situation, this study focuses on the impact of high-speed rail and innovation on the green economy and explores whether the flow of talents can moderate the promotion of technological development for green economic activities.

The construction of high-speed rail and the development of technology can promote the growth of a green economy, and this could be explained by the impact of knowledge creation consequences on green economic activities. When cities obtain more and more highly productive workers, the labor market in these cities will provide adequate support for green economic development. To better capture the flow of talents, the opening time of high-speed rail is used to represent the links between different cities, which also provides some options for talents to migrate from mega cities to secondary cities. The construction of high-speed rail can improve the knowledge share and the quality of technological update, so that a large amount of environmental benefits would be produced by green economic activities. It is worth noting that the flow of talents could be regarded as policy-oriented migration, and the impact of high-speed rail on the green economy was stronger during China’s 12th Five-Year Plan. Moreover, the green economy of cities with weak economic strength and low administration could not get adequate support from the outcomes of innovation, which may be due to the technical disadvantages of these cities.

Consistent with the theory of the green economy, this study explains how talent and technology have an impact on the green economy and discusses the moderating role of high-speed rail in the promotion of innovation to facilitate green growth. Our findings can help local governments pay attention to the spatial equilibrium distribution of talents and provide some new ideas to policymakers to achieve the goals of sustainable development.

### Recommendations and limitations

Green economy is a hot topic related to environmental fields and economic fields, and exploring how to promote the development of a green economy can help governments to achieve the goal of sustainable development, especially in developing countries. Our findings can enrich the literature on green growth and knowledge spillover. Based on the empirical analysis, this study has the following implications:

First, the impact of talents flow on the green economy could be changed by national development policies. When cities connect with high-speed rail, they will have an opportunity to obtain some highly productive workers. Achieving the spatial equilibrium distribution of talents should be considered with the implementation of government policy, which can also strengthen the promotion of technological update to a green economy.

Second, the outcomes of innovation have always been the driving factor of the green economy. Some cities with weak economic strength and low administration should invest more resources in the development of technology and cannot only rely on the migration of talents to obtain some existing technological outcomes. Secondary cities should obtain technological advantages through the construction of high-speed rail and focus on the impact of innovation in the green economy.

There are some limitations to this study. For the development of a green economy, we use green GDP to measure the development of a green economy in the environmental dimension, but this measurement may ignore the social benefits of this concept. In addition, the differences between cities may influence the empirical results, and some cities with economic and technological advantages will easily achieve the goal of green growth. In future research, we need to explore the driving factors of a green economy in similar cities and use more comprehensive measurement to represent the features of a green economy.

## Data availability statement

The data sources of this study are the Official Railway Service website of China (www.12306.cn), the China Urban Statistical Yearbook (https://www.mohurd.gov.cn/gongkai/fdzdgknr/sjfb/tjxx/jstjnj/index.html), the website of the National Bureau of Statistics of China (https://data.stats.gov.cn/), and the Chinese Research Data Services Platform Database (https://www.cnrds.com/).

## Author contributions

FC and WW: conceptualization. DK: methodology. XZ: software and visualization. JL: formal analysis. FC and DK: writing—original draft preparation. WW and JL: writing—review and editing. All authors have read and agreed to the published version of the manuscript.

## Footnotes

1
https://www.12306.cn/

2
https://data.stats.gov.cn/
